# Anti-cancer activity of Annexin V in murine melanoma model by suppressing tumor angiogenesis

**DOI:** 10.18632/oncotarget.16645

**Published:** 2017-03-29

**Authors:** Xuerui Zhang, Lina Huo, Haibo Jin, Yuheng Han, Jie Wang, Yanjun Zhang, Xinghuan Lai, Ziwei Le, Jing Zhang, Zichun Hua

**Affiliations:** ^1^ The State Key Laboratory of Pharmaceutical Biotechnology, School of Life Science, Nanjing University, Nanjing 210093, Jiangsu, China; ^2^ Changzhou High-Tech Research Institute of Nanjing University and Jiangsu Target Pharma Laboratories Inc., Changzhou 213164, Jiangsu, China; ^3^ The State Key Laboratory of Natural Medicines, China Pharmaceutical University, Nanjing 210009, Jiangsu, China

**Keywords:** Annexin V, apoptosis, necrosis, tumor angiogenesis

## Abstract

Annexin V, a protein with high affinity to phosphatidylserine (PS) in a calcium dependent manner, has been widely used to probe apoptosis. Annexin V in inhibiting engulfment of apoptotic cells by macrophages had been reported to increase the immunogenicity of tumor cells undergoing apoptosis. However, far less is known about its multiple properties, especially in cancer therapies. Here we found that Annexin V had a good anti-tumor activity in murine melanomaxenograft model. Treatment with Annexin V showed significant reduction in tumor size and remarkable tumor necrosis areas. The serum level of VEGF was downregualted by Annexin V both in normal mice and mice bearing tumor, suggesting that its new role on impeding tumor angiogenesis. *In Silico* analysis using Oncomine database, we also found the negative correlation of AnnexinV and VEGF both in skin and melanoma. The decreased Annexin V expression shows linearity relation with the elevated VEGF expression. These data provided a possibility that Annexin V can be used as a novel angiogenesis inhibitor in tumor therapy.

## INTRODUCTION

Annexin V is a well characterized member of the Annexin family and was originally discovered as an anticoagulant and antithrombotic protein [1]. The main biochemical characteristic of Annexin V is binding anionic phospholipids in a Ca^2+^-denpendent manner. Phosphatidylserine (PS) is confined to membrane leaflets facing the cytosol in the healthy cells, while translocated to the exofacial leaflet of the plasma membrane during apoptosis [2]. The high-affinity binding of Annexin V to PS has been broadly used for identifying apoptotic cells [3].

Apoptotic cells are poorly immunogenic because of their swift removal *in vivo* [4]. The role of Annexin V in inhibiting engulfment of apoptotic cells by macrophages had been proved by Heidi Kenis, etc [5]. Incubation of apoptotic cells with Annexin V prior to the immunization of mice significantly increased the immunogenicity of the cells undergoing apoptosis. It indicated that an impaired clearance of dying tumor cells can lead to tumor rejection and Annexin V leaded to an impaired clearance of apoptotic cells [6, 7]. Xenograft tumor models in Annexin V-deficient mice also confirmed that Annexin V rendered dead tumor cells immunogenic. Tumor cure appendages with dead tumor cells should be performed with Annexin V as an immune stimulator and could be combined with chemotherapy and irradiation therapy *in vivo*. Because immunogenic cell death forms should be induced *in vivo* by chemotherapeutic agents agents [8], ionizing irradiation [9], hyperthermia [10], treatment with Annexin V acts as immune activating agent [4].

Several recent studies have also indicated that exposure of PS occurs on vascular endothelium in solid tumors [11, 12]. PS is present on the luminal surface of vascular endothelial cells in various tumors, but not in normal tissues [13–15]. It suggested that Annexin V, as PS-recognizing protein, might be used for delivering cytotoxic drugs, coagulants for the selective destruction or imaging of vessels in solid tumors. PS-positive tumor endothelium for the most part seemed to be viable in the tumors and does not display markers of apoptosis, indicating that PS exposure is probably involved in other biological events. Angiogenesis is a fundamental step in the transition of tumors from a benign state to a malignant one. Tumors always secrete a plethera of growth factors, including VEGF, to the signaling cascades that culminate in pro-aniogenic events [16]. This caused our interest to study whether interference with the PS recognition by Annexin V is related to tumor angiogenesis.

In the present study, the efficacy of Annexin V for tumor growth suppression was examined in the B16F10 melanomaxenografts in mice. The treatment with Annexin V significantly retarded the tumor growth and showed increased necrosis in tumor tissues. More importantly, we found that Annexin V inhibited the angiogenesis by downregulating the level of VEGF. Using Oncomine database, we revealed that Annexin V expression was a linear negative correlation with VEGF expression. Furthermore, low expression of Annexin V in patients has a poor prognosis by Kaplan Meier plotter for meta-analysis. It suggested that Annexin V could be used as a potential molecule of anti-angiogenesis in tumor therapy.

## RESULTS

### Expression and purification of Annexin V

**Figure 1:**
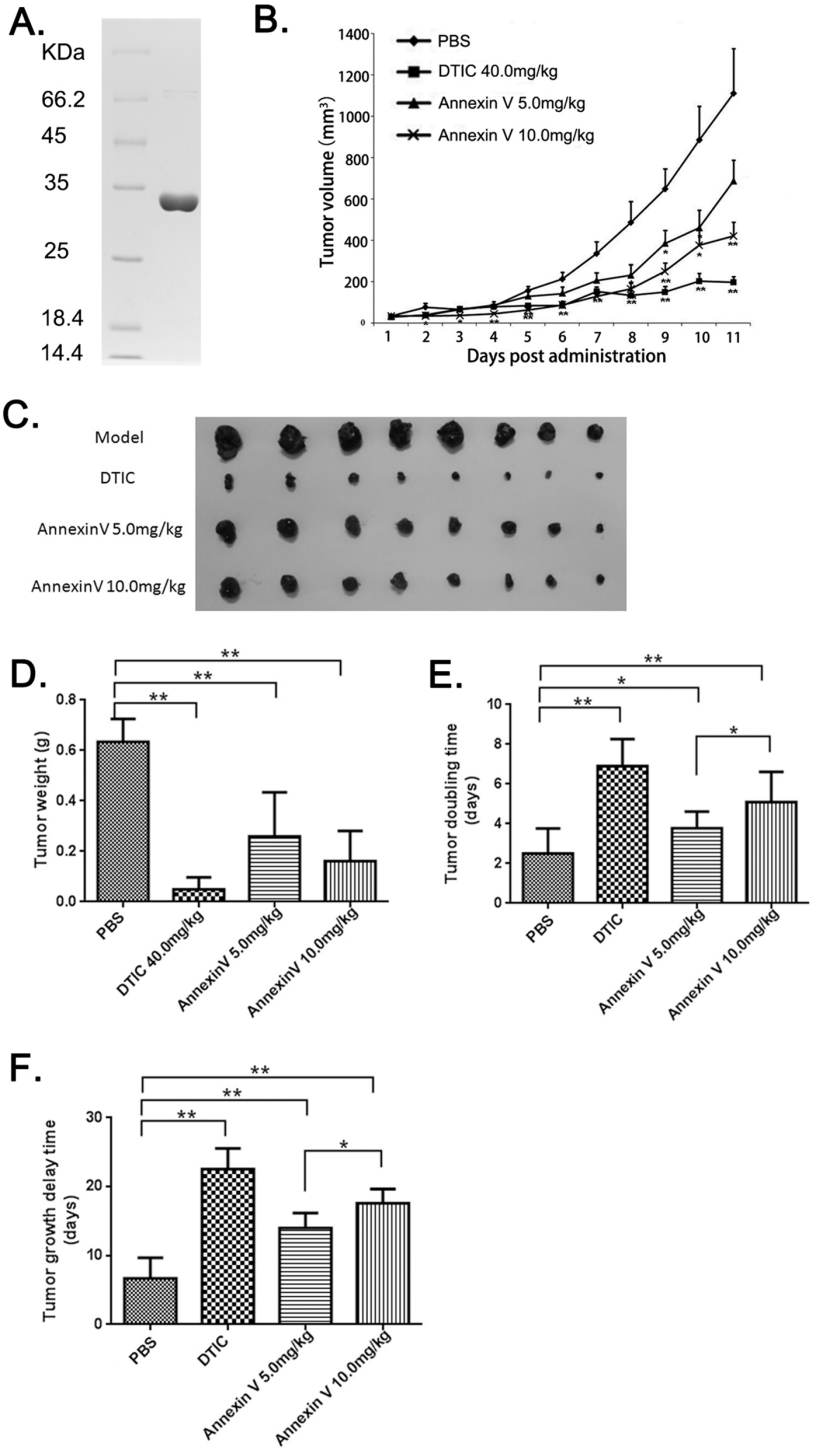
The anti-tumor effects of Annexin V in mice bearing B16F10 melanomas **(A)** Analyses of the purified Annexin V by SDS–PAGE on 12% resolving gel. The gel was stained with Coomassie blue R-250. Left lane: molecular-weight standard; Right lane: the purified rHV3. **(B)** Tumor volumes comparison among different groups (Mean ± SEM, n=8, **p<0.01 compared with PBS group). **(C)** B16F10 xenografts from different groups. **(D)** Tumor weights comparison among different groups (Mean ± SEM, n=8, **p<0.01 compared with PBS group). **(E)** Tumor doubling time comparison among different groups (Mean ± SEM, n=8, **p<0.01 compared with PBS group). **(F)** Tumor growth delay time comparison among different groups(Mean ± SEM, n=8, **p<0.01 compared with PBS group).

The protein Annexin V was expressed in *Escherichia coli BL21* and purified by our lab as described before [17]. The final purified product (~0.5 mg) was analyzed by SDS-PAGE. The result showed that the molecular weight of Annexin V was approximately 34 kDa and the purity was over 98% (Figure 1A), which was consistent with published paper [18].

### Annexin V suppressed tumor growth of B16F10 Xenografts in mice

Mice xenografts model with murine melanoma B16F10 was established. These four treatment groups were assigned to receive vehicle (i.e. PBS) as negative control, anticancer drug DITC as positive control, and two dosages of Annexin V in treatment. In the B16F10 model, tumor volumes were significantly smaller after Annexin V treatment as compared to PBS, and the inhibition of tumor growth by Annexin V was in a dosage-dependent manner (Figure 1B and 1C). The tumor weights in Annexin V treatment groups also showed remarkably reduced compared to the PBS group (Figure 1D). Tumor doubling time was 2.4 days for PBS controls, 3.8 days for 5 mg/kg annxin V and 5.1 days for 10 mg/kg Annexin V (p<0.05) and similar results were shown in tumor delay time compared with the controls (Figure 1E and 1F). Through regression analysis for the treatment effects on tumor growth, we observed administration of Annexin V, especially 10.0 mg/kg, could notably impede the tumor growth rate (Table 1). Overall, these results showed the tumor-inhibitory activity of Anenxin V.

**Table 1:** Regression analysis for treatment effects on tumor growth

Treatment	n	Growth curve^a^v(d)	R^2^	Tumor doubling time(d)^b^	Tumor growth delay time(d)^c^
PBS	8	ln(V) = 0.3474×d + 3.2983	0.98	2.5	9.4
DTIC 40 mg/kg	8	ln(V) = 0.1834×d + 3.4491	0.92	6.9	22.5
AnnexinV 5.0 mg/kg	8	ln(V) = 0.3125×d + 3.2922	0.98	3.8	14
AnnexinV 10.0 mg/kg	8	ln(V) = 0.287×d + 3.0596	0.97	5.1	16.7

^a^Regression growth curves summarize volume (V, mm3) dependence on time (d, days) from initial treatment, with correlation coefficients indicated by r.

^b^Tumor doubling time was derived from exponential growth curves.

^c^Growth delay was determined by assessing the time interval to 1000 mm^3^ compared with the PBS control.

### Administration of Annexin V with little toxicity

**Figure 2:**
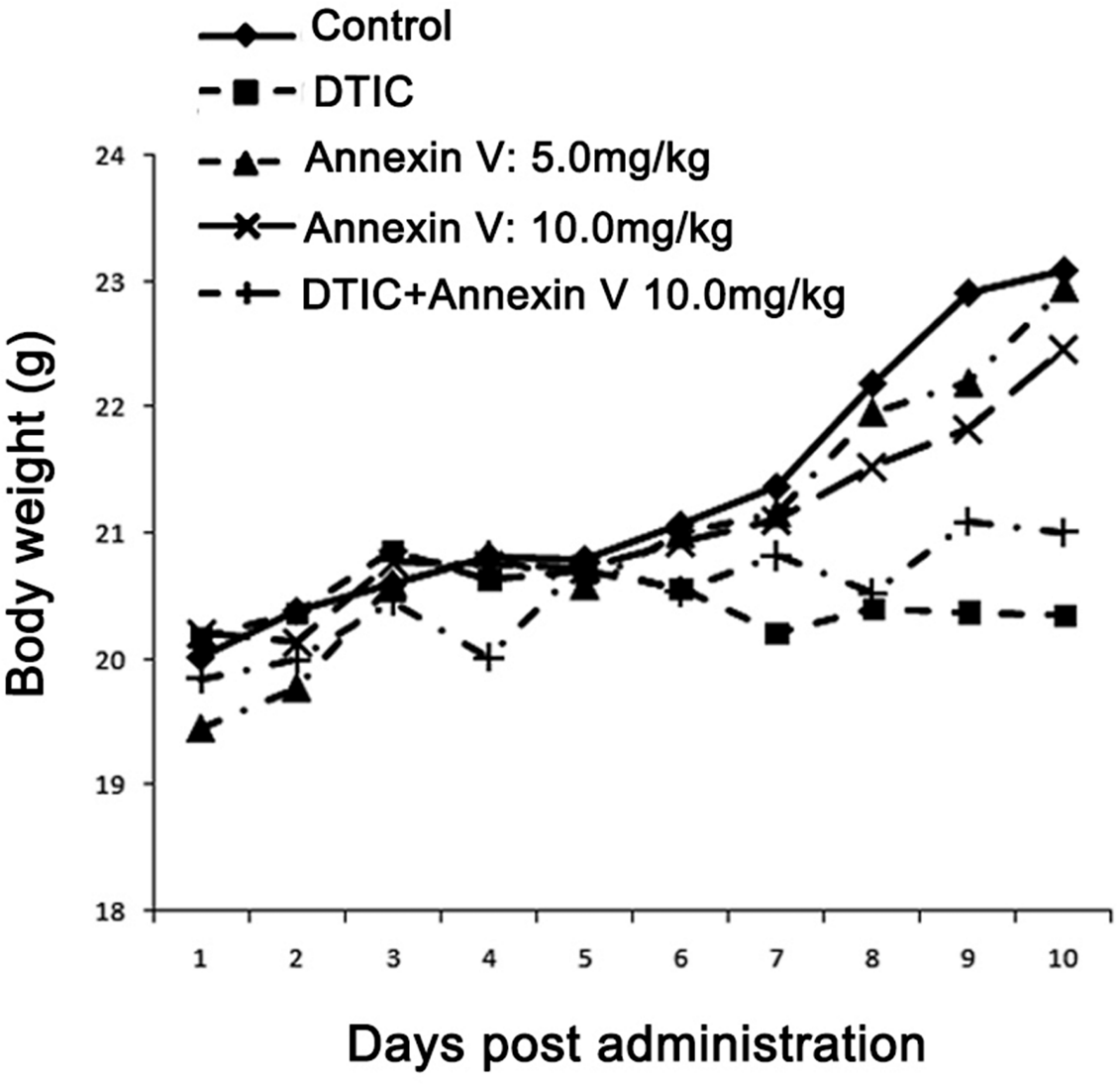
Body weight of mice bearing B16F10 melanomas Treatment with Annexin V of both 5.0 mg/kg and 10.0 mg/kg tended to keep body weights better than the positive control (DTIC).

When examining body weight of the mice bearing tumor, we observed a steady increase in body weight when treatment with PBS, and an approximately 10% loss in body weight for anticancer drug DITC treated mice in the model (Figure 2). Administration of Annexin V tended to keep the body weight better compared to a loss of body weight in the DTIC group, indicating that Annexin V has little toxicity.

### Annexin V induced tumor cell necrosis *in vivo*

**Figure 3:**
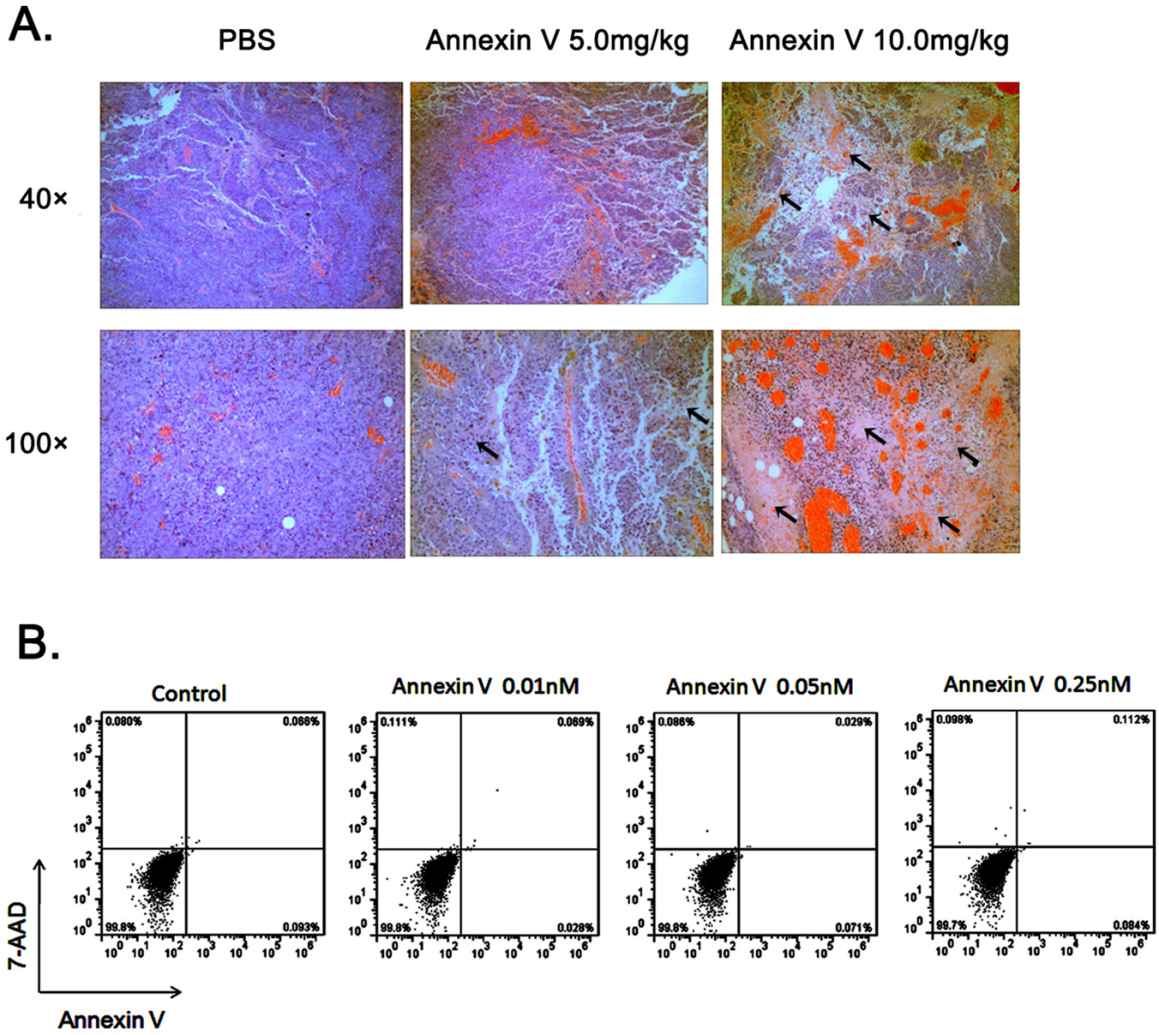
Administration of Annexin V promotes tumor necrosis *in vivo* **(A)** Histological analysis of tumor necrosis comparison among different groups. black arrows, necrotic cells. **(B)** Annexin V does not induce cell death of B16F10 tumor cells. B16F10 cells were treated with different concentrations of Annexin V and assayed by flow cytometry with staining of Annexin V-FITC and 7-AAD. Early/primary apoptotic cells (Annexin V^+^/7-AAD^−^), late/secondary apoptotic cells (Annexin V^+^/7-AAD^+^) and necrotic cells (Annexin V^−^/7-AAD^+^) were distinguished.

The tumor slices obtained from the mice in each group were examined histologically by hematoxylin and eosin (H&E) staining. Histological correlations showed a markedly heterogeneity in B16F10 tumor with a core comprised of cellular debris consistent with necrosis. Compared to PBS controls, the pronounced necrosis in the groups of Annexin V treatment was increased (Figure 3A). The most obvious necrosis was observed in mice with high dose of Annexin V 10 mg/kg in administration. It's worth mentioning that Annexin V itself does not induce cell death of B16F10 tumor cells (Figure 3B).

### Annexin V targeted tumor vessels and inhibited angiogenesis by downregulating the level of VEGF

**Figure 4:**
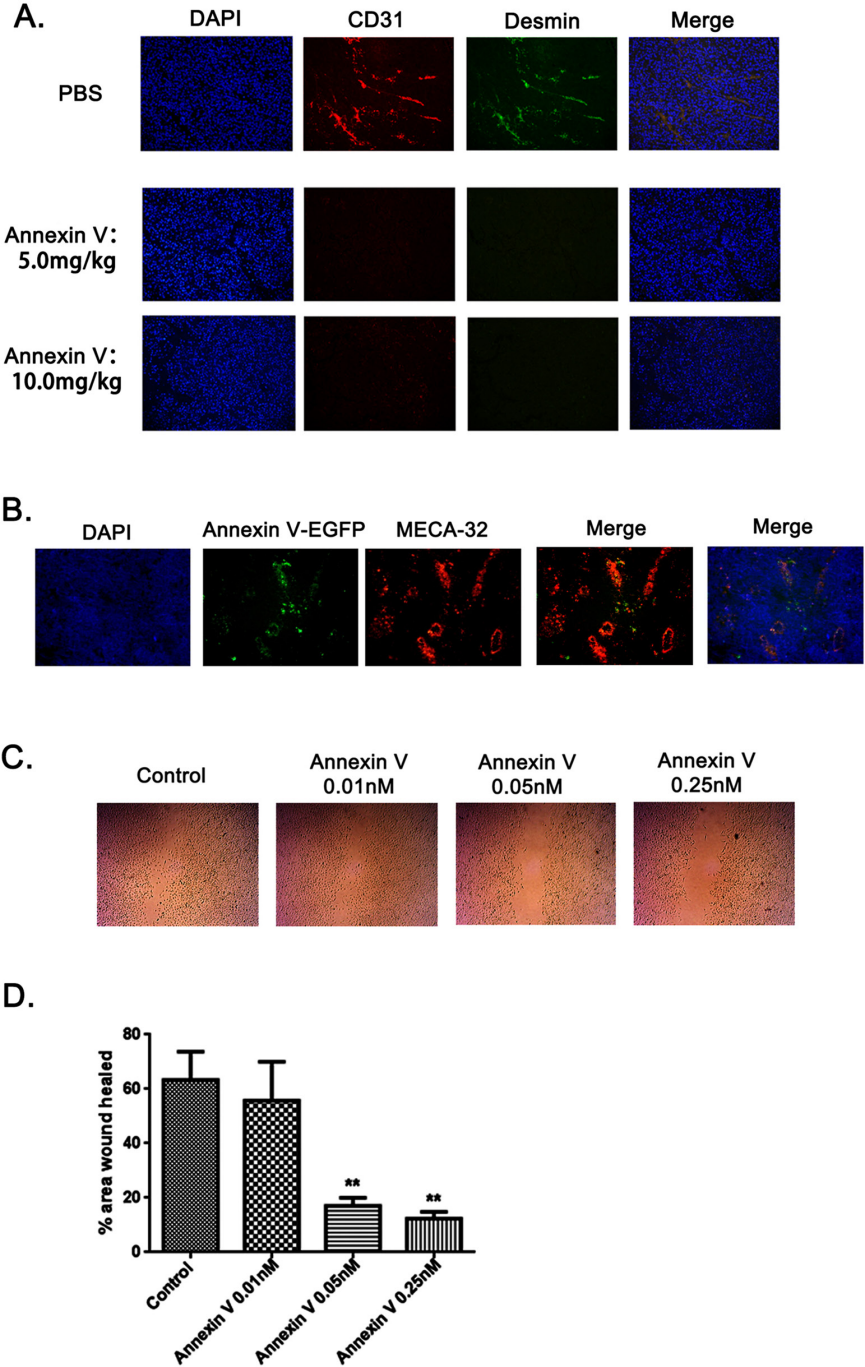
Annexin V targeted tumor vessels and inhibited tumor angiogenesis **(A)** Fluorescence images of tumor blood vessels. Vescular staining (immunofluorescent) was performed and showed co-localization of CD31 (red) and Desmin (green) in PBS-treated samples. Far few vescular endotheliums were detected in tumor sections treated with Annxin V (5.0 mg/kg and 10.0 mg/kg). **(B)** Annexin V targeted tumor vessels. Annexin V-EGFP was injected i.v. to mice bearing B16F10 solid tumors. After 30 min, frozen sections of tumor tissues were analyzed for the localization of Annexin V-EGFP and blood vessels identified by MECA-32 antibody. **(C)** *In vitro* endothelial cell wound healing assay. HUVEC cells treated with Annexin V migrated slower than its negative control in a dose-dependent manner. **(D)** Statistic analysis of HUVEC cell wound healing experiment (Mean ± SEM, n=3, **p<0.01 compared with negative control).

PS exposure on tumor vascular endothelium has been well documented [11, 12, 19, 20]. Since tumor necrosis is commonly due to impaired blood supply [21], we next examined whether Annexin V affects tumor angiogenesis. Sections from B16F10 tumors were assessed for vessel density by immunofluorescence using antibody against desmin and murine CD31, a marker of endothelial cells. Vascular desity was significantly decreased in tumors treated with Annexin V compared to PBS controls (Figure 4A), implying that Annexin V impedes tumor growth, at least in part, by inhibiting angiogenesis. To explore its possible mechanism, Annexin V-EGFP was injected by tail vein to mice bearing B16F10 solid tumor, and 30 min later, sections of tumor tissue were observed for the localization of Annexin V-EGFP under fluorescence microscope (Figure 4B). Bloodvessels were identified from their positive stainingby the endothelial cell antibody, MECA 32, on serial sections. Vascular endothelium in the tumors showed distinct membrane staining, and Annexin V was present on the luminal surface of capillaries and vessels in the tumors. Next we examined the effect of Annexin V on endothelium cell motility by wound healing assay in the HUVEC cells. Annexin V treatment effectively hindered the healing process in a dose-dependent manner compared with control group (Figure 4C). Quantivative analysis also revealed a significant reduced in the cell migration by Annexin V (Figure 4D). Collectively, these results proposed a possibility for antitumor acitivity of Annexin V based on its effect on tumorangiogenesis.

**Figure 5:**
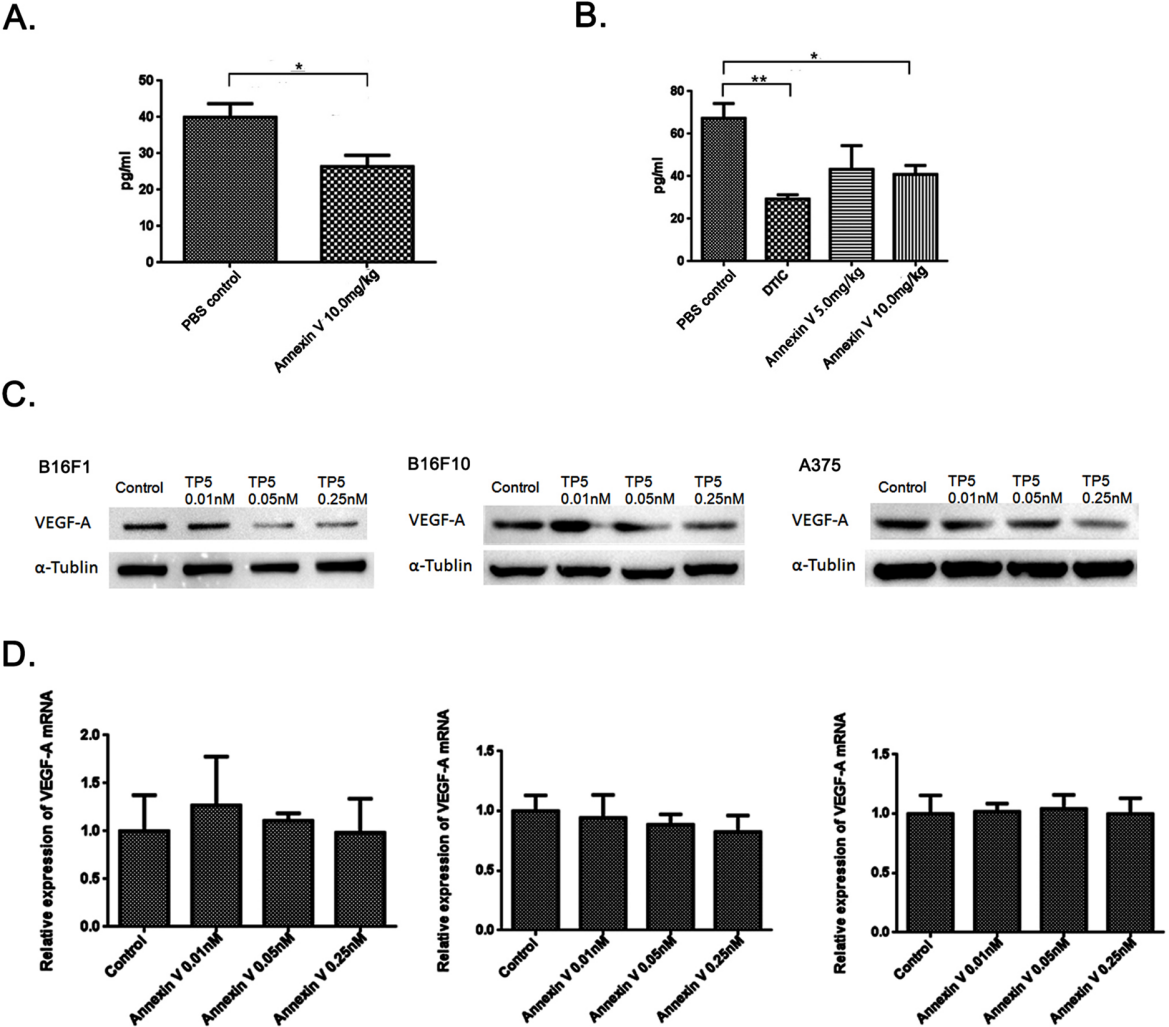
Annexin V downregulates VEGF expression at protein level, not at transcriptional level **(A)** Serum levels of VEGF in normal mice injected with 10.0 mg/kg Annexin V detected by ELISA (Mean ± SEM, n=8, **p<0.01 compared with PBS group). **(B)** Serum levels of VEGF in mice bearing B16F10 melanomas injected with Annexin V (5.0 mg/kg and 10.0 mg/kg) detected by ELISA (Mean ± SEM, n=8, **p<0.01 compared with PBS group). **(C)** The effect of Annexin V on the VEGF expression of B16F1 cells, B16F10 cells and A375 cells, respectively, at protein level monitored by western blot analysis. **(D)** The effect of Annexin V on the VEGF expression at transcriptional level determined by qPCR.

To further address the mechanism of lower microvascular densities by Annexin V, we detected thevascular endothelial growth factor (VEGF) levels in the serum from mice bearing tumors. As expected, the secretion of VEGF in the serum was inhibited by Annexin V treatment in mice bearing tumor (Figure 5A). More interestingly, Annexin V also reduced the serum levels of VEGF in normal mice under physiological conditions (Figure 5B), suggesting a potential regulation of VEGF by Annexin V *in vivo*. Then we tested the effect of Annexin V on VEGF expression at protein level in melanoma cell lines B16F1, B16F10 and A375. Annexin V at dose form 0 to 0.25 nM caused a dose-dependent decrease in the expression of VEGF in both mouse and human melanoma cells (Figure 5C). Next VEGF mRNA was examined by qPCR assays and no significant difference were observed in these cells treated with Annexin V (Figure 5D), indicating the effect of Annexin V on VEGF expression not at transcriptional level, probably at posttranslational processing.

### A negative correlation between annexin V and VEGF expression in melanoma

**Figure 6:**
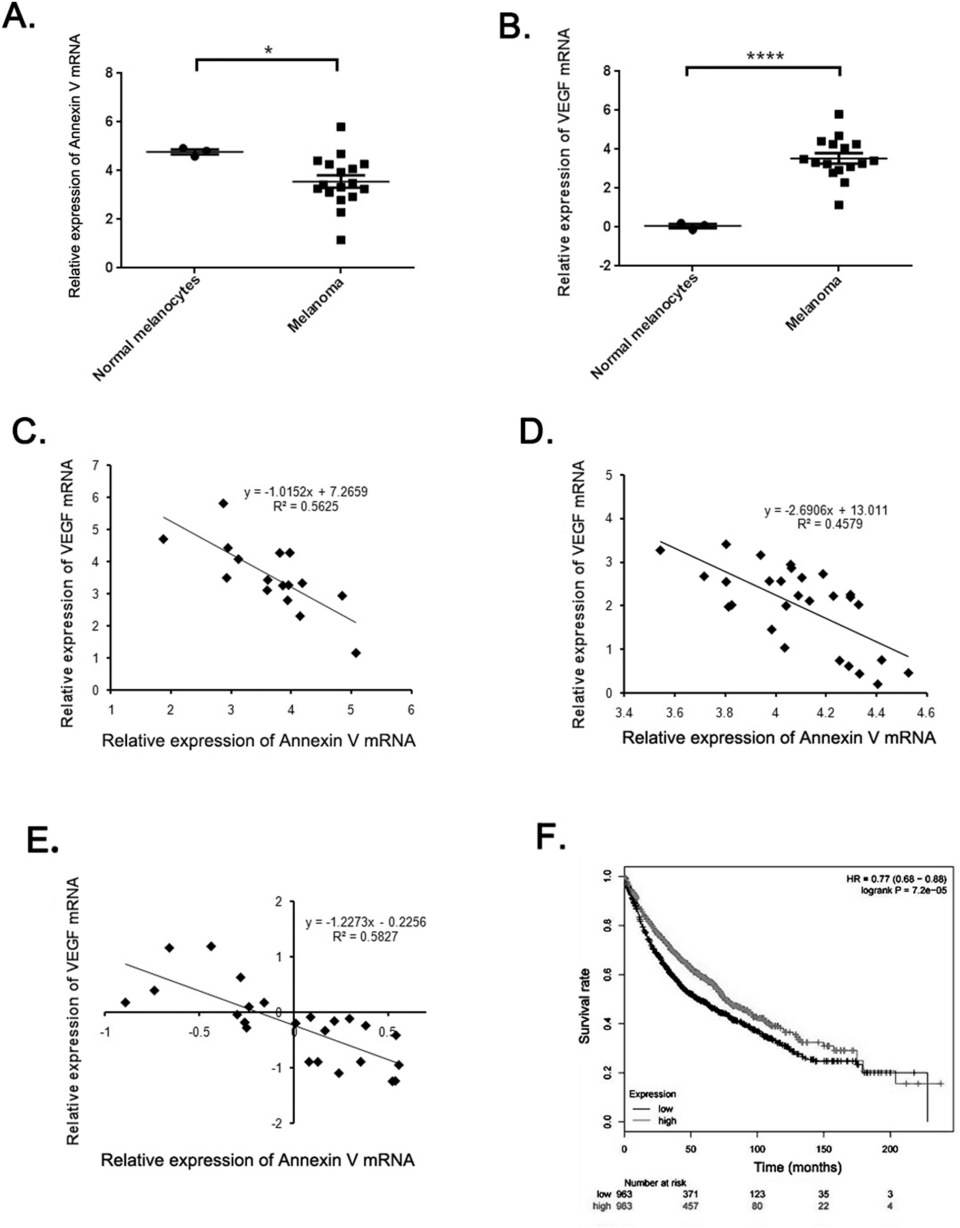
The negative correlation between Annexin V and VEGF expression in melanoma **(A-B)** Analysis of Annexin V and VEGF expression levels in normal melanocytes and most melanomas using Harlin melanoma dataset. The results showed a significant decrease of Annexin V levels and a remarkable rise of VEGF levels in most melanomas, compared to the normal group. (Mean ± SEM, n=8, *p<0.05, ****P<0.0001 compared with PBS group). **(C-E)** Regression analysis of correlation between Annexin V and VEGF expression levels using Harlin, Xu, and Hoek melanoma datasets, respectively. **(F)** The impact of Annexin V expression levels on the survival rates of melanoma patients using Kaplan Meier plotter. The results suggested that a high expression level of Annexin V tended to indicate an improved prognosis, and vice versa.

Based on the above finding, we raised the question regarding the correlation between expression of Annexin V and VEGF in melanoma. To gain the information of Annexin V and VEGF expression in human melanoma, we performed analysis of published patient's data using Oncomine (http://www.oncomine.org), a free online bioinformatic resource including clinical mRNA array data of different genes from different patients all over the world. In Harlin melanoma dataset with 52 samples [22], Annexin V expression levels were downregulated in most melanoma tisssues (P<0.05) (Figure 6A), and increased VEGF expression was observed in human melanoma compared to normal skin cells (P<0.001) (Figure 6B). Through the regression analysis, the two genes showed the negative correlation, the decreased Annexin V expression was linearity relation with the elevated VEGF expression in human melanoma (Figure 6C). Similar results reconfirmed in other two melanoma datasets, Xu melamona and Hoek Melanoma (Figure 6D and 6E). Using Kaplan Meier plotter, another free online tool for meta-analysis based biomarker assessment, the result revealed that low expression of annexin V in patients was correlated with a worse survival compared with high expression. Collectively, these finding indicate that down-regulated annexin V predicts a poor prognosis and is closely correlated with tumor angiogenesis, suggesting that Annexin V can be used as a novel angiogenesis inhibitor in tumor therapy.

## DISCUSSION

Annexin V binds to PS, which is exposed at the surface of apoptotic cells and has broadly used as a probe to measure apoptosis *in vivo* and *in vitro* [23]. Now the efficacy of new anti-tumor compounds to induce cell death in solid tumors can be investigated by radiolabelled Annexin V *in vivo* imaging [24]. Strategies to enhance the immunogenicity of tumors are urgently needed. Annexin V binds with high affinity to PS on the surface of apoptotic and necrotic cells and thereby impairs their uptake by macrophages. The impaired clearance of apoptotic tumor cells induced by Annexin V may contribute to the immune-activating tumor microenvironment. Exposed PS is not only a feature of apoptotic cells, but is also present on the surface of microvesicles and angiogenic endothelium [25–27]. Oncogenic EGFR was reported to shed from cancer cells as cargo of membrane microvesicles, which can interacted with surfaces of other cells. Annexin V could also block the oncogene-containing microvesicles exchange between tumor cells and impede tumor angiogenesis through blocking MVs transmission of angiogenic oncogenes between tumor cells and endothelial cells.

In this study, we reconfirmed the anti-tumor activity of Annexin V (Figure 1). Previous published paper by us reported a chimeric protein Annexin V-TRAIL with higher efficacy in inhibiting tumor growth than TRAIL both *in vivo* and *in vitro* [17]. It indicated that Annexin V, in addition to interfere cell death, was very likely to be involved in other events. Accumulating data suggest that PS tends to be specifically exposed on vescular endothelial cells in all tumors so far examined including orthotopically grown, likely ascribed to oxidative stresses present in the tumor microenvironment [11, 28]. We hypothesized that Annexin V will play an important role on angiogenesis through binding the exposed PS residue on vescular endothelial cells. By immunofluorescence assay, we observed the suppression of tumor angiogenesis with the treatment of Annexin V, and further ELASA assay showed the secretion of VEGF was also blocked by Annexin V (Figure 4). The results indicated that Annexin V might be a promising tumor therapeutic agent or angiogenesis inhibitor. Consistent with reduced tumor angiogenesis, more necrosis areas were observed in the tumor tissues from mice of Annexin V group (Figure 3).

*In vivo* induction of immunogenic tumor cells can be applied as tumor vaccine and this immunogenicity might be further increased by coupling Annexin V to the tumor cells. As suggested by the findings reported here, Annexin V may play an important role during tumor angiogenesis, and affect a more response to this angiogenic growth factor VEGF.

## MATERIALS AND METHODS

### Production and purification of Annexin V

Annexin V was produced and purified according to the protocol described before [17]. In brief, the plasmid PET28a-Annexin V was constructed and stored by our lab. Recombinant Annexin V was expressed in Escherichia coli BL21 (DE3) cells (Novagen) and purified by metal affinity chromatography on Zn-sepharose and anion exchange chromatography on DEAE-Sepharose. All of the solutions were prepared with sterile, endotoxin-free water. Protein concentrations were determined using BCA Protein Assay Reagent (Galen Biopharm). After purification, Annexin V was analyzed by SDS-PAGE under reduced conditions. The concentrations of resolving gel and stacking gel were 12% and 8%, respectively. Molecular weight markers from Bio-rad Company were used.

### Xenograft experiments

Murine melanoma cellline B16F10 were purchased from the American Type Culture Collection. Cells were stored and recovered from cryopreservation in liquid nitrogen and cultured in DMEM (Wisent, Canada) medium plus 10% FBS (Invitrogen, USA), 50 mg/ml streptomycin and 50 U/ml penicillin and cultured in 5% CO_2_ humidified atmosphere. Female C57/B6 mice (6-8 weeks old) were purchased from from Beijing Animal Centre, and maintained in pathogen-free conditions. 1×10^5^ B16F10 cells in 100 μl PBS were injected into the mid-right flank of mice, which developed tumors in 7-10 days with the size of approximately 50-100 mm^3^. For every experiment, the mice were randomized into 4 groups (8 mice per group) and intraperitoneally injected with PBS, 5.0 mg/kg Annexin V, 10.0 mg/kg Annexin V, 40.0 mg/kg dacarbazine (DTIC, for positive control) daily for continuous 13 days. Tumor volume was measured every day after the initial injection with calipers and determined asmm3 using the equation: A×B^2^×0.52 [29, 30], where A was the length (mm) and B was the width (mm). Tumor doubling time refers to the time for a tumor to double in volume and tumor growth delay time is the time interval to reach 1000 mm^3^ compared with the PBS control group [17]. Animal care and use were performed strictly in accordance with the ethical guidelines by the Nanjing University Animal Care and Use Committee, and the study protocol was approved by the local institution review board.

### H&E staining and immunofluorescence

H&E staining and immunofluorescence was carried out as described before [31]. In brief, tumor samples at study end point were fixed in 4% paraformaldehydeor 16-24 h and transferred to 70% ethanol before paraffin embedding. Three-micrometre sections were generated, and one section from each sample was stained with hematoxylin and eosin (H&E).

For immunostaining, sections were deparaffinised, rehydrated and antigen retrieval performed with citric acid (pH 6.0) in an 850W microwave. For CD31 immunostaining, antigen retrieval was performed with proteinase K at 37°C. Non-specific protein binding was blocked with 10% donkey serum (Sigma). Sections were incubated with primary antibodies for 1 h at room temperature or overnight at 48C. The primary antibodies used were against Desmin (1:50; BD Biosciences) and CD31 (1:50; BD Biosciences). Then, sections were incubated with appropriate AlexaFluor-conjugated secondary antibodies for 1 h at room temperature and counterstained with DAPI.

### ELISA

Serum levels of VEGF of mice from different groups were detected using commercial ELISA kit according to the manufacturer's instructions (R&D Systems).

### QPCR and western blot

Total RNA was isolated with Trizol regeant (Invitrogen, USA) and then reverse transcribed using the qPCR RT kit (Toyobo, Japan). Quantitive real time PCR was performed with a StepOne/StepOnePlus^TM^ Real-Time PCR System (Applied Biosystems) usingSYBR Green PCR Master mix according to the manufacturer's instructions (Roche) with specific primers: murine VEGF-A, 5′-TTACTGCTGTACCTCCACC-3′ (Forward); 5′-ACAGGACGGCTTGAAGATG-3′ (Reverse); human VEGF-A, 5′-GAAGTGGTGAAGTTCATGGATGTC-3′ (Forward); 5′-CGATCGTTCTGTATCAGTCTTTCC-3′ (Reverse); murine Annexin V, 5′-TCCTCCTTCAG GCGAATAGA-3′ (Forward); 5′-TGTCCCAAAGATG GTGATGA-3′ (Reverse); human Annexin V, 5′-CTTGGGCACAGATGAGGAGAGCA-3′ (Forwad); 5′-AAGCCGAGAGGGTTTCATCAGAGC-3′ (Reverse); murine β-actin, 5′-GCTTCTAGGCGGACTGTTAC TGA-3′ (Forward); 5′-GCCATGCCAATGTTGTC TCTTAT-3′ (Reverse); humanβ-actin, 5′-CGGCATC GTCACCAACTG-3′ (Forward); 5′-GGCACACGC AGCTCATTG-3′ (Reverse).

For Western blot analysis, Cells were washed twice with ice-cold PBS aftercollection and suspended in a lysisbuffer [50 mM Tris-HCl (pH 7.4), 250 mM NaCl, 0.5%Triton-X 100, 50 mM NaF, 2 mM EDTA, 1 mM Na3VO4, anda protein inhibitor cocktail] for 30 min and then centrifuged(13,000 g, 10 min, 4°C). Supernatant was separated using 12% SDS-PAGE andtransferred to PVDF membranes (Millipore, Bedford, US). After that, the membranes were blocked in non-fat 5% milk and then incubated with primary antibodies aganst VEGF-A (Cell signaling) and α-Tublin (Abgent), followed by suitablesecondary antibody conjugated with horseradish peroxidase. Reactive bandswere detected with an ECL Western Blot Detection Kit (CST) and visualized with a Tanon Imager program (Tanon, China). The intensities of the scanned bands were normalized to the β-actin signal.

### *In vitro* endothelial cell wound healing assay

HUVEC cells were seeded in 6-well plates at 1 × 10^5^ cells/well to form confluent monolayers. The monolayers were incubated in the absence of serum for 12 h and wounded in a line across the well with a 200-μl standard pipette tip. The wounded monolayers were then washed twice with serum-free media to remove cell debris and incubated with different concentrations of Annexin V. Migration into the open wound was photographedat different time points until the scratch was nearly closed.

### Statistical analysis

Unless otherwise stated, error bars indicate SEM, and P values of <0.05 after a two-tailed *t* test are denoted by an asterisk in the figures.
